# Association of the difference between cystatin C- and creatinine-based estimated glomerular filtration rate with cerebral small vessel disease: A large prospective cohort study

**DOI:** 10.1016/j.tjpad.2025.100190

**Published:** 2025-04-24

**Authors:** Zhiming Li, Fei Wang, Jincheng Liu, Benbo Xiong, Han Wang, Zijie Wang, Xiao Hu, Qi Li

**Affiliations:** a Department of Neurology, The Second Affiliated Hospital of Anhui Medical University, Hefei, China; b Department of Neurology, Xiangyang Hospital of Traditional Chinese Medicine, Xiangyang, Hubei, China; c Department of Clinical Medicine, The Second School of Clinical Medicine, Anhui Medical University, China

**Keywords:** Cerebral small vessel disease, White matter hyperintensity, Estimated glomerular filtration rate, Blood pressure, Dementia

## Abstract

**Background and objective:**

It remains unclear whether the difference between the estimated glomerular filtration rate based on cystatin C and creatinine (eGFRdiff) is associated with cerebral small vessel disease (CSVD). We investigated the correlation of eGFRdiff with SCVD and further evaluated the mediating role of blood pressure.

**Methods:**

This prospective cohort study included 35,590 neurologically healthy participants at baseline (2006 to 2010) from the UK Biobank. eGFRdiff is divided into two indicators: absolute difference (eGFRabdiff) and ratio (eGFRrediff) based on the calculation between cystatin C and creatinine. CSVD was assessed by calculating white matter hyperintensity volume (WMHV) from T2-FLAIR brain MRI scans (conducted between 2014 and 2021), with values normalized to intracranial volume and log-transformed. Multiple linear regression models and mediation analysis was used to evaluate the associations of eGFRdiff with WMHV.

**Results:**

Participants with negative eGFRabdiff had higher WMHV (β = 0.07, 95 % confidence interval [CL] = 0.04 ∼ 0.10), while participants with positive eGFRabdiff had smaller WMHV (β = -0.05, 95 %CL = -0.09 ∼ -0.02), compared to midrange eGFRabdiff group. Meanwhile, participants with eGFRrediff ≤ 0.7 had higher WMHV compared with participants with eGFRrediff > 0.7 (β = 0.08, 95 %CL = 0.01∼ 0.15) .In addition, hypertension mediated the associations between eGFRdiff and WMHV (12.6 % ∼13.2 %).

**Conclusion:**

eGFRdiff was independently associated with WMHV. Our findings suggested that monitoring eGFRdiff has potential benefits in identifying the burden of CSVD in the general population in future.

**Clinical Perspective**

What was known?

Intraindividual differences between estimated glomerular filtration rate (eGFR) based on cystatin C (eGFRcys) and creatinine (eGFRcr) have been associated with a range of adverse clinical outcomes. However, the clinical implications of these differences (eGFRdiff) for the cerebral small vessel disease (CSVD), a major contributor to cognitive decline, stroke remains unclear.

What is new?

In this large prospective cohort study comprising 35,590 UK Biobank participants, we found those with large negative values of eGFRdiff had a higher burden of CSVD.

The analysis of hypertension as a mediator in the relationship between eGFRdiff and CSVD burden revealed that it accounted for 13 % of the effect.

3.In the stratified analysis, no significant interactions were observed between the eGFRdiff and other factors, except for race. This suggests that the effect of the eGFRdiff on the outcome may be influenced or modified by racial differences, while other factors do not appear to alter this relationship.

What are the clinical implications?

Our findings highlight that eGFRdiff could be a more dependable marker for assessing CSVD burden. In clinical practice, monitoring the intraindividual eGFRdiff may optimize the risk clinical assessment and management of CSVD, potentially aiding in the early prevention of stroke and dementia.

Alt-text: Unlabelled box

## 1 Introduction

Cerebral small vessel disease (CSVD) is a common and significant pathology associated with aging and a major contributor to cognitive decline, stroke, and other neurological disorders [1]. White matter hyperintensity (WMH) is increasingly being utilized to monitor the progression of CSVD, as it is a crucial magnetic resonance imaging (MRI) marker of CSVD [2]. The relationship between systemic vascular health and CSVD is well-established, with hypertension being one of the most significant modifiable risk factors contributing to disease progression [3]. However, the precise mechanisms linking vascular health markers with CSVD remain incompletely understood, particularly with respect to renal function markers.

The brain and kidneys share a profound connection, anchored in their analogous anatomical structures and hemodynamic characteristics of small vessel [4]. Functional changes in the kidney are associated with CSVD, particularly in community-based populations with cardiovascular disease (CVD) [5,6]. Additionally, inflammatory processes, and oxidative stress following changes in kidney function are considered to contribute to the risk of vascular damage and endothelial dysfunction [4]. The estimated glomerular filtration rate (eGFR), usually measured by serum cystatin C or creatinine, is a commonly used biomarker in clinical practice to evaluate renal function and microvascular inflammation [7]. However, cystatin C-based eGFR (eGFRcys) and creatinine-based eGFR (eGFRcr) differs significantly in some individuals (such as the elderly, sarcopenia, and people with less physical activity), suggesting that the large differences may serve as markers of health status [8,9]. Previous studies have shown that eGFRdiff is associated with adverse events such as CVD, frailty, and mortality, but whether it is associated with CSVD remains unclear [[9], [10], [11], [12]].

This study aims to address this gap by investigating the association between the difference in eGFRcys and eGFRcr (eGFRdiff) and CSVD, measured by white matter hyperintensity volume (WMHV), in a large cohort of neurologically healthy individuals from the UK Biobank. We hypothesize that participants with greater discrepancies between cystatin C and creatinine-based GFR estimates will demonstrate higher WMHV, reflecting a higher burden of CSVD [13]. Furthermore, given the established role of blood pressure in both renal and cerebral vascular health, we also evaluate the mediating effect of blood pressure status on the relationship between eGFRdiff and WMHV.

## 2 Methods

### 2.1 Study population

**Fig. 1:**
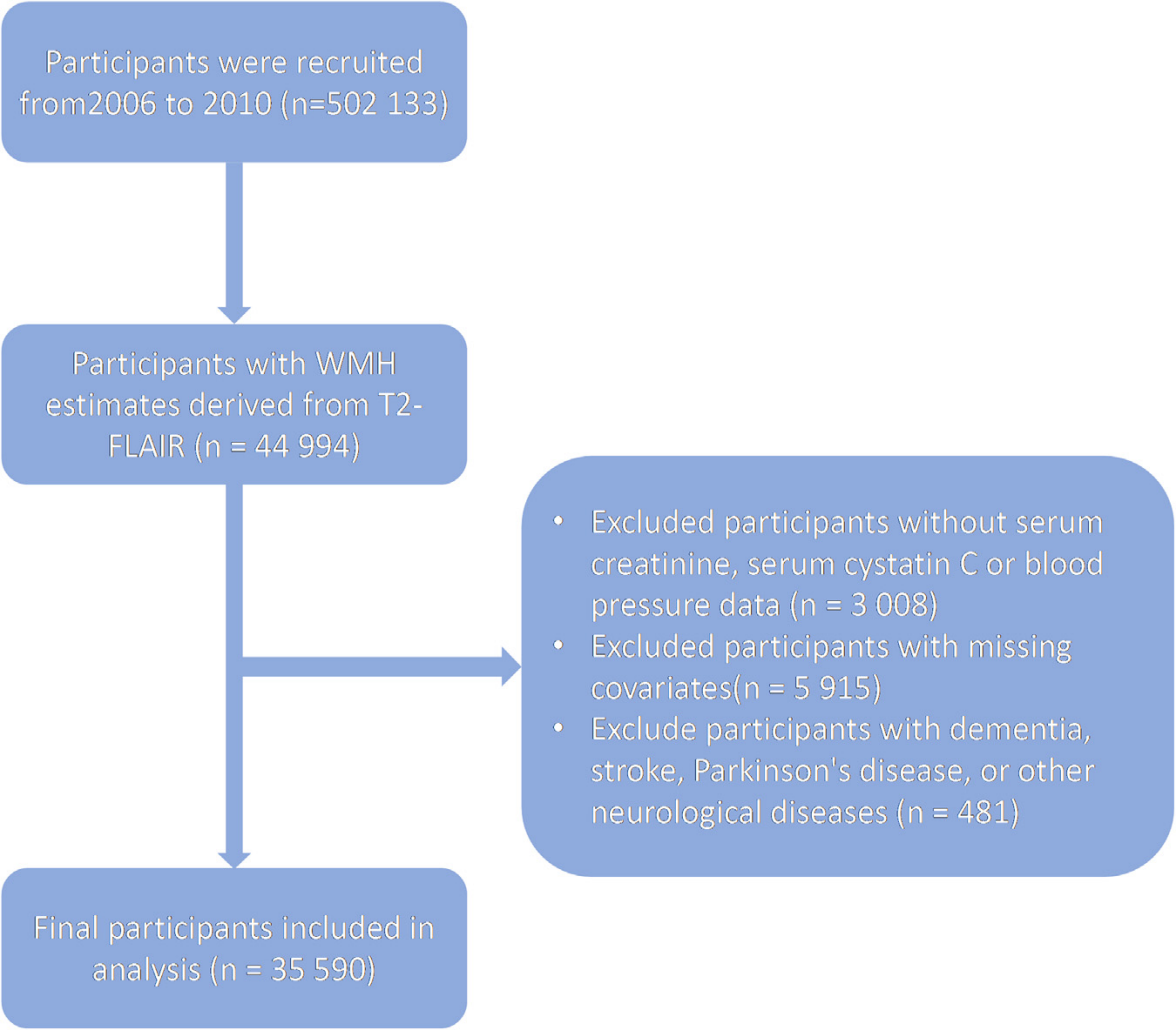
**Flow diagram describing sample selection**.

UK Biobank is a large-scale, prospective cohort study that aims to reveal the impact of environmental, genetic and lifestyle factors on human health through extensive collection and analysis of data. This project recruited approximately 500,000 participants aged 40 to 69 from across the UK between 2006 to and 2010.The design and methods of this study have been described in detail previously [14]. We initially enrolled 44,994 participants from the UK Biobank MRI program into the study. Furthermore, we excluded participants with missing baseline data on serum creatinine and cystatin as well as other covariates. Participants diagnosed with stroke, dementia, Parkinson's disease, and or other neurological diseases (including multiple sclerosis, any other chronic degenerative, neurologic disorder, brain cancer, brain abscess, aneurysm, cerebral palsy, encephalitis, head or neurologic injury, nervous system infection, trauma) were further excluded [13]. Finally, 35,590 participants were included in our analysis. A flowchart outlining the participant selection process is shown in Fig. 1.

### 2.2 Main exposure

Serum creatinine and cystatin were measured in the UK Biobank using the enzymatic analysis and the latex enhanced immunoturbidimetric analysis on a Beckman Coulter AU5800 and a Siemens ADVIA 1800, respectively. The 2012 Chronic Kidney Disease Epidemiology Collaboration (CKD-*EPI*) cystatin C equation and 2021 race-free CKD-*EPI* equations were utilized to compute eGFRcys and eGFRcr, respectively [7,15]. eGFRdiff included the absolute difference between eGFR (eGFRabdiff) and relative difference between eGFR (eGFRrediff). eGFRabdiff was defined as eGFRcys minus eGFRcr, while eGFRrediff defined as eGFRcys/eGFRcr. The eGFRabdiff was further stratified by three categories: negative eGFRabdiff (< −15 mL/min/1.73 m^2^), midrange eGFRabdiff (−15 to 15 mL/min/1.73 m^2^), and positive eGFRabdiff (≥ 15 mL/min/1.73 m^2^), while the eGFRrediff was categorized into two groups: <0.7, and ≥0.7 [10,16].

### 2.3 Outcome

In our study, we used total white matter hyperintensity volume (WMHV) as a biomarker of total burden of CSVD [13]. Participants underwent 3T MRI scanning at the UK Biobank Imaging Centre. The standardized scanning protocol includes multimodal imaging sequences to provide comprehensive brain structural and functional data [17]. Brain Intensity Abnormality Classification Algorithm (BIANCA) was used to calculate the WMHV from images on T1 and T2-FLAIR sequences [17]. Intracranial volume was calculated using the image processing pipeline developed by UK Biobank. To correct for differences in intracranial volume among different individuals, WMHV was normalized by intracranial volume (WMHVnorm).

### 2.4 Covariates

In the UK Biobank, information on demographic characteristics and lifestyle was obtained using a touch screen questionnaire. Demographic characteristic includes age, sex, ethnicity and Townsend deprivation index (TDI). To protect the privacy of participants, we categorized race into white and non-white in this study. TDI was calculated based on the proportions of unemployment, non-car ownership, non-house ownership and household overcrowding among all participants. Alcohol and drinking history were categorized as never and ever. Anthropometric measurements (height, weight, and body composition) and blood pressure were measured by a trained nurse. In addition, serum lipid profile and high-sensitivity C-reactive protein (hs-CRP) were also quantitatively measured at baseline. BMI≥30 was defined as obesity. Hypertension was defined as Systolic blood pressure (SBP) ≥140mmhg or diastolic blood pressure (DBP) ≥90mmhg. Hyperlipidemia was defined as Triglycerides ≥ 6.2mmol/L. Diabetes was self-reported during a verbal interview with a trained nurse at the assessment center.

### 2.5 Statistical analysis

The baseline characteristics were described according to categories of eGFRabdiff and eGFRrediff. Continuous variables were presented as mean and standard deviation, while categorical variables were presented as numbers and percentages. The differences between the groups were compared using the Kruskal-Wallis rank sum test and Wilcoxon rank sum test for categorical variables and the Pearson's Chi-squared test for continuous variables. We performed the Anderson-Darling test to check the normal distribution of continuous variables. We log-transformed WMHVnorm to correct its positively skewed distribution. To reduce the influence of potential confounding factors, we constructed several multivariate linear regression models to accurately investigate the effect of eGFRdiff on WMHVnorm. Model 1 was unadjusted. Model 2 was adjusted for age, sex, ethnicity and TDI. Model 3 was further adjusted for diabetes, hypertension, hyperlipidemia, obesity, smoking history, drinking history, hs-CRP, HDL-cholesterol (HDL-C), total cholesterol (TC) and eGFRcys.

To investigate the dose-response relationship between eGFRdiff and WMHVnorm, we used a restricted cubic spline (RCS) regression model with 3 knots at the 10th, 50th, and 90th percentiles of the eGFRdiff distribution and nonlinearity was tested with the likelihood ratio test. We further performed mediation analysis to determine whether and to what extent the relationship between eGFRdiff and WMHVnorm was mediated by BP.

We performed several sensitivity analyses to validate these findings. First, we performed subgroup analyses to explore the impacts of eGFRabdiff and eGFRrediff on WMHVnorm in different populations. Subgroup analyses were stratified by age, sex, ethnicity, drinking history, alcohol history, hypertension, diabetes and hyperlipidemia. Second, we further adjusted for muscle mass for statistical analysis. Third, we repeated the statistical analysis by replacing eGFRcr and eGFRcr-cys with eGFRcr respectively. Fourth, we defined the eGFRrediff replication statistics in different ways to verify stability. In addition, we also excluded individuals with baseline chronic kidney disease and conducted statistical analysis. Finally, we explored the correlation between eGFRdiff and WMHVnorm at different time periods. All statistical analyses were performed using R software (version 4.3.1). All statistical tests were two sided, and the critical level of significance was set at 0.05.

## 3 Result

### 3.1 Baseline characteristics

Table 1 shows baseline characteristics of participants in our study. Among the 35,590 participants we included, the mean age was 54.98 ± 7.53, and 18,671(52 %)were women. Among all participants, 6530(18 %) had negative eGFRabdiff, 26,467(74 %) had midrange eGFRabdiff, and 2593(7.3 %) had positive eGFRabdiff. There were 762(2.1 %) participants with eGFRrediff < 0.7, and 34,828(98 %) participants with eGFRrediff > 0.7. The mean eGFRabdiff and mean eGFRrediff were -4 ± 13 and 0.97 ± 0.15 mL/min/1.73 m^2^ at baseline. Participants with a large negative eGFR difference (both eGFRabdiff and eGFRrediff) had a higher proportion of females, as well as a greater prevalence of obesity, smoking history, diabetes, hypertension, and hyperlipidemia (*p* < 0.05). Additionally, these participants exhibited a poorer metabolic profile, characterized by elevated total cholesterol (TC), higher C-reactive protein (CRP) levels, and lower high-density lipoprotein cholesterol (HDL-C).

**Table 1:** Baseline characteristics of participants by category of eGFRdiff in UK Biobank.

		eGFRabdiff (ml/min/1.73m^2^)				eGFRrediff		
**Characteristic**	Overall, *N* = 35,590(100 %)^1^	< −15, *N* = 6530(18 %)^1^	−15∼15, *N* = 26,467(74 %)^1^	>15, *N* = 2593(7.3 %)^1^	P-Value^2^	<0.7, *N* = 762(2.1 %)^1^	≥0.7, *N* = 34,828(98 %)^1^	P-Value^3^
Age (years)	54.98 ± 7.53	55.84 ± 7.31	54.94 ± 7.56	53.27 ± 7.51	<0.001	57.46 ± 6.85	54.93 ± 7.54	<0.001
Gender (%)					<0.001			<0.001
Female	18,671(52 %)	4700(72 %)	13,076(49 %)	895(35 %)		603(79 %)	18,068(52 %)	
Male	16,919(48 %)	1830(28 %)	13,391(51 %)	1698(65 %)		159(21 %)	16,760(48 %)	
Race/ethnicity (%)					<0.001			0.7
Others	1003(2.8 %)	195(3.0 %)	675(2.6 %)	133(5.1 %)		20(2.6 %)	983(2.8 %)	
White	34,587(97 %)	6335(97 %)	25,792(97 %)	2460(95 %)		742(97 %)	33,845(97 %)	
TDI	−1.89 ± 2.72	−1.59 ± 2.86	−1.95 ± 2.68	−2.05 ± 2.63	<0.001	−1.39 ± 2.96	−1.90 ± 2.71	<0.001
Serum Creatinine (umol/L)	72 ± 14	64 ± 11	73 ± 13	88 ± 12	<0.001	64 ± 11	72 ± 14	<0.001
Serum Cystatin C (mg/L)	0.87 ± 0.13	0.97 ± 0.13	0.85 ± 0.12	0.79 ± 0.09	<0.001	1.12 ± 0.17	0.87 ± 0.12	<0.001
eGFRcr (ml/min/1.73m^2^)	96 ± 14	102 ± 11	96 ± 13	81 ± 11	<0.001	101 ± 12	96 ± 14	<0.001
eGFRcys (ml/min/1.73m^2^)	92 ± 14	80 ± 12	94 ± 13	103 ± 11	<0.001	66 ± 9	93 ± 14	<0.001
eGFRcr-cys (ml/min/1.73m^2^)	109 ± 15	110 ± 15	110 ± 16	101 ± 12	<0.001	103 ± 16	109 ± 15	<0.001
eGFRabdiff	−4 ± 13	−23 ± 7	−2 ± 8	22 ± 7	<0.001	−35 ± 7	−3 ± 12	<0.001
eGFRrediff	0.97 ± 0.15	0.78 ± 0.06	0.98 ± 0.08	1.29 ± 0.13	<0.001	0.65 ± 0.05	0.98 ± 0.14	<0.001
TC (mmol/l)	5.73 ± 1.07	5.86 ± 1.12	5.71 ± 1.06	5.62 ± 1.04	<0.001	5.83 ± 1.18	5.73 ± 1.07	0.041
HDL-C(mmol/l)	1.48 ± 0.38	1.44 ± 0.37	1.48 ± 0.38	1.49 ± 0.37	<0.001	1.38 ± 0.37	1.48 ± 0.38	<0.001
CRP (mg/l)	2.06 ± 3.58	3.02 ± 4.55	1.88 ± 3.35	1.50 ± 2.52	<0.001	4.43 ± 5.74	2.01 ± 3.50	<0.001
HbA1c (%)	5.35 ± 0.46	5.41 ± 0.49	5.34 ± 0.46	5.33 ± 0.42	<0.001	5.48 ± 0.51	5.35 ± 0.46	<0.001
Obesity (%)					<0.001			<0.001
Yes	6475(18 %)	2035(31 %)	4141(16 %)	299(12 %)		341(45 %)	6134(18 %)	
No	29,115(82 %)	4495(69 %)	22,326(84 %)	2294(84 %)		421(55 %)	28,694(83 %)	
Smoking Status (%)					<0.001			<0.001
No Smoking	21,735(61 %)	3746(57 %)	16,322(62 %)	1667(64 %)		391(51 %)	21,344(61 %)	
Smoking	13,855(39 %)	2784(43 %)	10,145(38 %)	926(36 %)		371(49 %)	13,484(39 %)	
Drinking Status (%)					<0.001			<0.001
No Drinking	863(2.4 %)	228(3.5 %)	579(2.2 %)	56(2.2 %)		35(4.6 %)	828(2.4 %)	
Drinking	34,727(98 %)	6302(97 %)	25,888(98 %)	2537(98 %)		727(95 %)	34,000(98 %)	
Diabetes (%)					<0.001			<0.001
No	34,582(97 %)	6287(96 %)	25,761(97 %)	2534(98 %)		714(94 %)	33,868(97 %)	
Yes	1008(2.8 %)	243(3.7 %)	706(2.7 %)	59(2.3 %)		48(6.3 %)	960(2.8 %)	
Hypertension (%)					<0.001			<0.001
No	28,225(79 %)	4924(75 %)	21,176(80 %)	2125(82 %)		485(64 %)	27,740(80 %)	
Yes	7365(21 %)	1606(25 %)	5291(20 %)	468(18 %)		277(36 %)	7088(20 %)	
Hypercholesterolemia (%)					<0.001			<0.001
No	8484(24 %)	1294(20 %)	6473(24 %)	717(28 %)		142(19 %)	8342(24 %)	
Yes	27,106(76 %)	5236(80 %)	19,994(76 %)	1876(72 %)		620(81 %)	26,486(76 %)	
WMHV (mm^3^)	5177 ± 6855	5883 ± 7752	5085 ± 6670	4337 ± 6126	<0.001	6930 ± 8961	5138 ± 6797	<0.001
Normalized WMHV	0.004 ± 0.005	0.004 ± 0.005	0.003 ± 0.005	0.003 ± 0.004	<0.001	0.005 ± 0.006	0.004 ± 0.005	<0.001
Logit normalized WMHV	−6.18 ± 1.03	−6.06 ± 1.04	−6.20 ± 1.03	−6.36 ± 1.00	<0.001	−5.86 ± 1.01	−6.19 ± 1.03	<0.001

1 mean (IQR) for continuous; n (%) for categorical.

2 Kruskal-Wallis rank sum test; Pearson’s Chi-squared test.

3 Wilcoxon rank sum test; Pearson’s Chi-squared test.

#### 3.1.1 eGFRdiff and the burden of CSVD

**Fig. 2:**
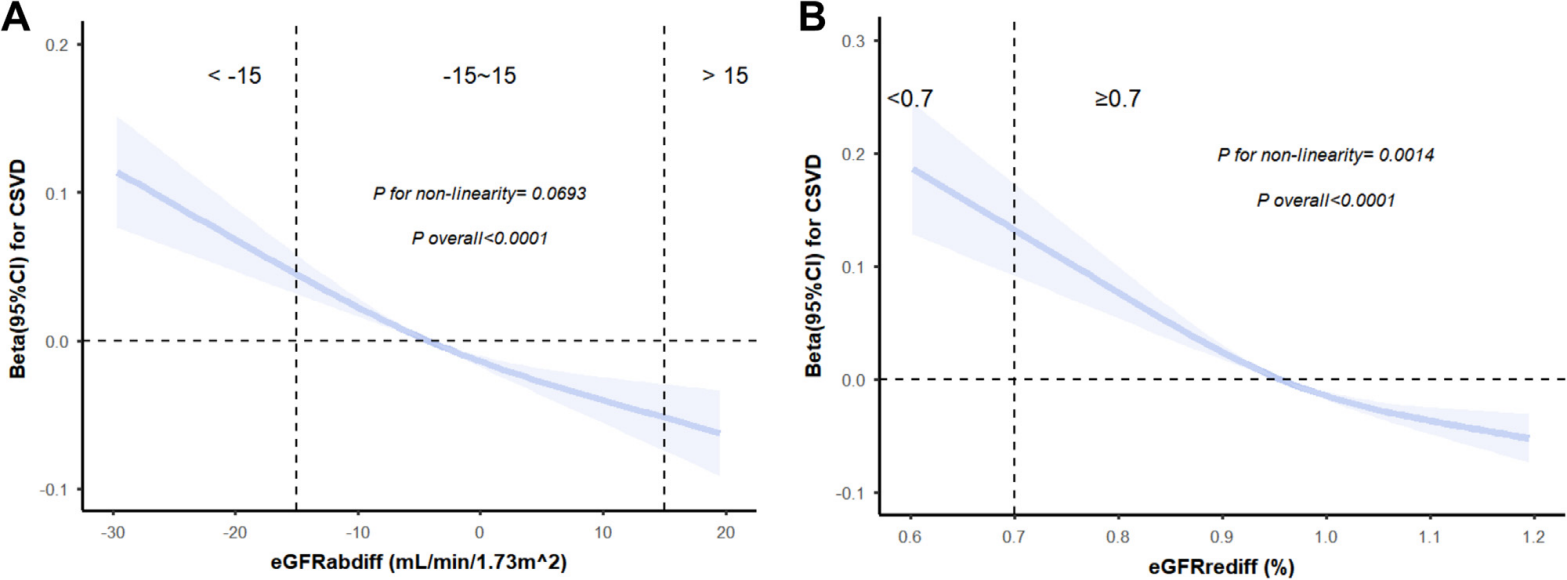
**Dose-response relationship of eGFRabdiff (A) and eGFRrediff (B) with the burden of CSVD.** Restricted cubic spline was used to explore nonlinear associations, with three knots fifixed at the 10th, 50th, and 90th percentiles for all smooth curves.The beta was derived using linear regression, which controlled for adjusted for age, gender, race and TDI, obesity, smoking status, drinking status, diabetes, hypertension, hypercholesterolemia, TC, HDL-C, CRP and eGFRcys. Abbreviations: eGFRabdiff, absolute difference between estimated glomerular filtration rate based on cystatin C and creatinine; eGFRrediff, ratio difference between estimated glomerular filtration rate based on cystatin C and creatinine; CSVD, Cerebral Small Vessel Disease; CI, confidence interval.

Lower eGFRdiff was associated with WMHVnorm in all multivariate analyses adjusted for confounders. Compared to participants with midrange eGFRabdiff, participants with negative eGFRabdiff showed a dangerous effect on WMHVnorm in the final adjusted model (β=0.07,95 %CL=0.04∼0.10) while participants with positive eGFRabdiff showed a protective effect on the WMHVnorm (β=−0.05, 95 %CL= −0.09∼−0.02) (Table 2). RCS analysis demonstrated a linear dose-response relationship between eGFRrediff and white matter hyperintensity volume (overall *P* < 0.001 ; P for non-linear= 0.069) (Fig. 2).

**Table 2:** Association between eGFR difference and Cerebral Small Vessel Disease.

	Group								
	Model1			Model2			Model3		
Characteristic	Beta	95 %CI^1^	p-value	Beta	95 %CI^1^	p-value	Beta	95 %CI^1^	p-value
**eGFRabdiff**									
−15∼15	1.00 (Ref.)			1.00 (Ref.)			1.00 (Ref.)		
<−15	0.14	0.11, 0.17	<0.001	0.11	0.08, 0.13	<0.001	0.07	0.04, 0.10	<0.001
>15	−0.16	−0.20, −0.12	<0.001	−0.06	−0.10, −0.03	0.003	−0.05	−0.09, −0.02	0.003
**eGFRrediff**									
≥0.7	1.00 (Ref.)			1.00 (Ref.)			1.00 (Ref.)		
<0.7	0.32	0.25, 0.40	<0.001	0.18	0.11, 0.24	<0.001	0.08	0.01, 0.15	0.018

1 CI = Confidence Interval Abbreviations: eGFRabdiff, absolute difference between cystatin- and creatinine-based estimated glomerular filtration rate; eGFRrediff, relative difference between cystatin- and creatinine-based estimated glomerular filtration rate. Model 1: non-adjusted model. Model 2: adjusted for age, gender, race and TDI. Model 3: Model 2 plus obesity, smoking status, drinking status, diabetes, hypertension, hypercholesterolemia, TC, HDL-C, CRP and eGFRcys.

Compared to participants with eGFRrediff≥0.7, participants with eGFRrediff <0.7 was associated with higher WMHVnorm (β=0.08, 95 %CL=0.01∼0.15) (Table 2). RCS analysis further indicated a nonlinear dose-response relationship between eGFRrediff and white matter hyperintensity volume (overall *P* < 0.0001; P for non-linear = 0.0014) (Fig. 2).

### 3.2 Mediation analysis

**Fig. 3:**
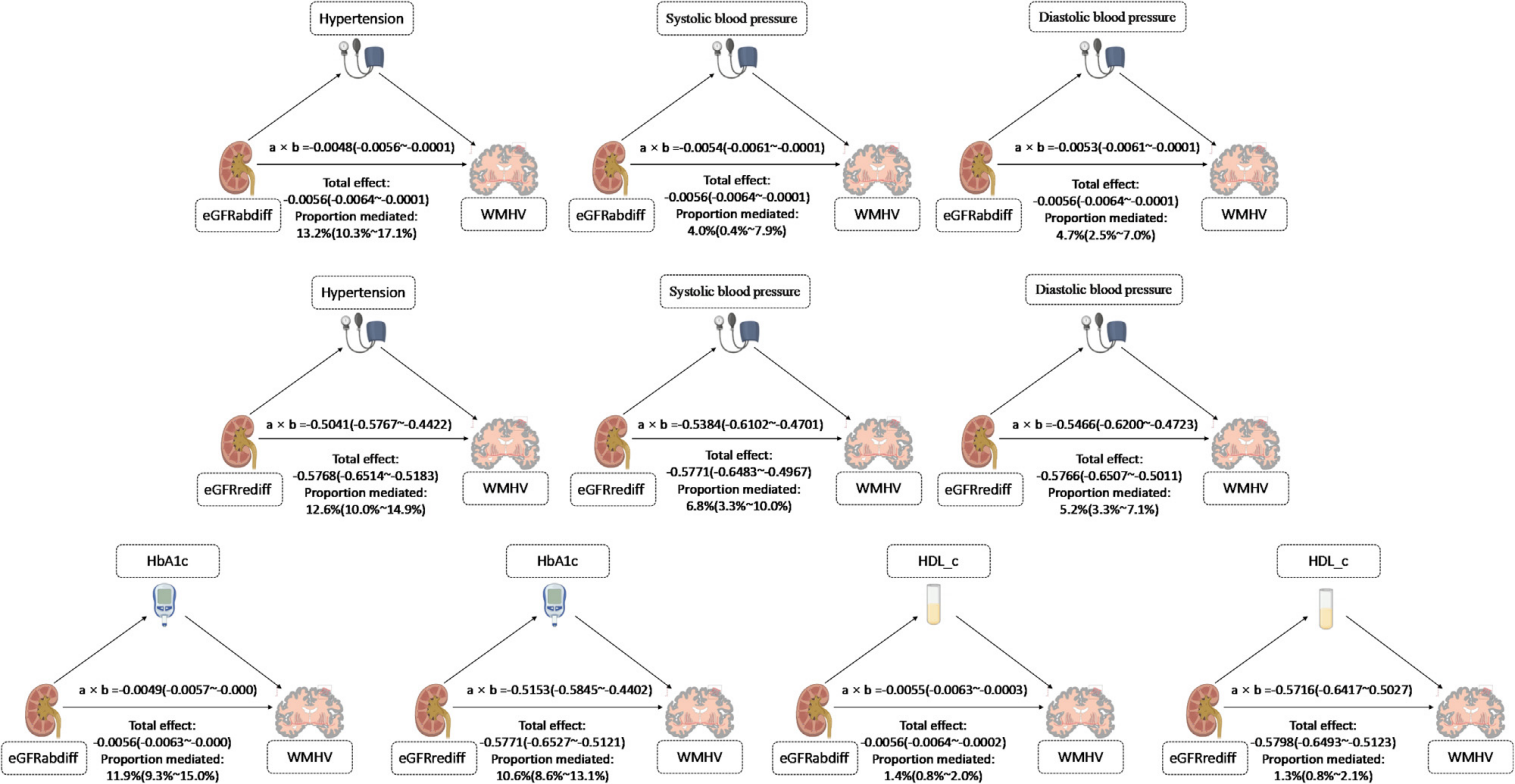
**Stratified analyses of the associations of eGFRabdiff (A) and eGFRrediff (B) with the burden of CSVD.** The beta was derived using linear regression, which controlled for adjusted for age, gender, race and TDI, obesity, smoking status, drinking status, diabetes, hypertension, hypercholesterolemia, TC, HDL-C, CRP and eGFRcys. P for interaction <0.05.

In our results, BP mediates the effect of eGFRdiff on WMHVnorm (Fig. 3). Specifically, we observed that the association of eGFRabdiff with WMHVnorm was significantly mediated by DBP, hypertension, and the indirect effect (IE) was −0.0053 (−0.0061∼−0.0001), −0.0048 (−0.0056∼−0.0001), with a proportion mediated of 4.7% and 13.2 %. In addition, our results showed that the association of eGFRrediff with WMHVnorm was also significantly mediated by SBP, DBP and hypertension, and the IE was −0.5384 (−0.6102∼−0.4701), −0.5466 (−0.6200 ∼−0.4723), −0.5041 (−0.5767∼−0.4422), with a proportion mediated of 6.8 %, 5.2 %, and 12.6 %.

### 3.3 Sensitivity analysis

**Fig. 4:**
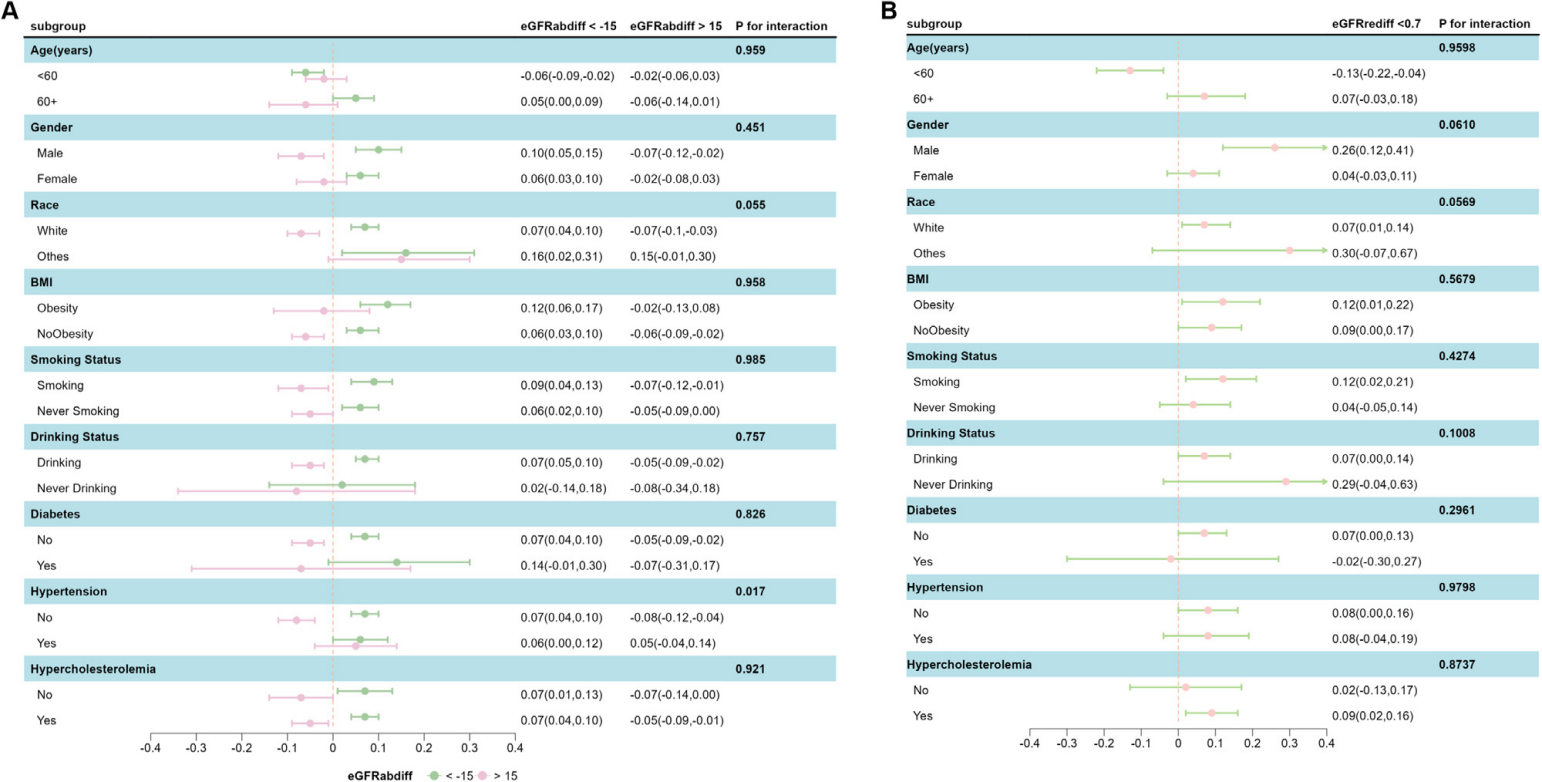
**Mediation efrects of blood pressure on the association between eGFRdiff and the burden of CSVD**.

Subgroup analysis was performed according to age, gender, BMI, smoking, alcohol consumption, race, blood lipids, and blood glucose status to identify potential influencing factors, but no significant interactions were found (Fig. 4). However, among individuals without hypertension, a greater positive eGFRabdif was significantly associated with lower WMHVnorm; this protective association was diminished in individuals with hypertension.When further adjusting for muscle mass, eGFRcr, and eGFRcr-cys, we found that the association between eGFRdiff and WMHVnorm remained significant, except for the association between eGFRdiff and WMHVnorm, which lost statistical significance when adjusting for eGFRcr and eGFRcr-cys (**Supplementary Table 1 and 2**). Furthermore, the association of eGFRrediff with WMHVnorm defined according to different classifications remained significant (**Supplementary Table 3**). We further excluded patients with chronic kidney disease at baseline and found that the association between eGFRdiff and WMHVnorm remained significant, except that eGFRrediff lost statistical significance when adjusting for model 3 (**Supplementary Table 4**). Additionally, we explored the associations between eGFRdiff over different time intervals and WMHVnorm. We found that the association between eGFRdiff and WMHVnorm remained significant; however, the protective effect associated with greater positive values of eGFRabdiff was no longer present (**Supplementary Table 5**).

## 4 Discussion

In this study, we investigated the relationship between eGFRdiff and CSVD in a large scale prospective cohort of neurologically healthy population. We observed a negative correlation between baseline eGFRdiff and WMHV, significantly mediated by blood pressure levels. This relationship was further influenced by race, with the association between eGFRdiff and WMHV being more pronounced among white individuals. These findings highlight the importance of monitoring eGFRdiff as part of a comprehensive risk stratification strategy for patients at risk of CSVD.

WMHV is a neuroimaging biomarker used to evaluate cerebrovascular health and is widely recognized to be associated with an increased risk of dementia and stroke [1,2]. Although WMHV is currently primarily attributed to ischemic injury, the precise neurovascular mechanisms underlying its development remain unclear [18]. Considering the anatomical and hemodynamic similarities between renal and cerebral vasculature, inflammation and oxidative stress resulting from renal dysfunction may concurrently impair endothelial function in both renal and cerebral vessels and ultimately resulting in a greater burden of WMH [4]. To date, several studies have revealed a significant association between renal function and WMHV in different specific populations. Among 2526 middle-aged and older participants in the Rotterdam Study, lower eGFRcr and lower eGFRcys were both positively associated with higher white matter lesion volume [4,19]. Other studies have also shown that both renal dysfunction and cystatin C levels are risk factors for white matter damage [20]. However, there may be significant differences between eGFRcys and eGFRcr in the same individual, reflecting various aspects of health status, such as muscle and fat mass, activity levels, and chronic inflammation [8,9].

Our study firstly reported that baseline eGFRdiff (both eGFRrediff and eGFRabdiff) was inversely correlated to WMHV. The associations remained robust even after adjusting for common risk factors and confounders, which suggested that a significantly negative eGFRdiff might serve as a unique predictor for unique predictor for burden of WMHV. Our findings were consistent with previous studies linking eGFRdiff to adverse cardiovascular events. In the Systolic Blood Pressure Intervention Trial (SPRINT), negative eGFRdiff was found to be associated with a higher risk of frailty, major adverse cardiovascular events (MACE), and mortality [9]. Another study reported a significant association between negative eGFRdiff and an increased risk of diabetic microvascular complications (DMCs) [10]. Additionally, other large cohort studies have also demonstrated that large negative eGFRdiff is significantly linked to an increased risk of atrial fibrillation and heart failure [11,21]. Additionally, analogical dose-response relationship patterns were observed between baseline eGFRdiff and the future burden of WMHV. More importantly, in our study, even after adjusting for eGFRcr or eGFRcys, the associations between eGFRdiff and WMHV remained robust, which suggested that these associations may be explained by non-GFR determinants rather than by a more accurate measurement of renal function. Building on previous research, effective screening of eGFRdiff in populations may provide valuable information for risk stratification and early intervention.

Subgroup analysis of this study showed that there was an interaction between eGFRabdiff and blood pressure status (*PP* < 0.05), indicating that blood pressure status and eGFRabdiff jointly affected WMHV. Our findings are consistent with previous studies, in which hypertensive population appear to have a higher burden of WMHV [22,23]. However, subgroup analyses adjusted for age, sex, BMI, smoking, hypertension, and diabetes were conducted to identify potential influencing factors, but no significant interactions were observed.

The potential mechanisms underlying the association between eGFRdiff and WMHV burden remain unclear. Consistent with previous studies, our results showed that participants with larger negative eGFRdiff were more likely to be obese, smokers, and have higher prevalence rates of hypertension and diabetes [24,25]. These are all widely recognized risk factors for WMH. Considering that some studies also support that antihypertensive treatment can delay the progression of WMH, this study further explored and confirmed the mediating role of blood pressure status between eGFRdiff and WMHV burden [26]. In addition, in the absence of extrarenal factors affecting cystatin C or creatinine, eGFRcys less than 60 % or 70 % of eGFRcr is defined as Shrunken pore syndrome [16]. Recent studies have shown that certain proteins associated with atherosclerosis, inflammation, and endothelial damage, such as interleukin-6, MCP-3, and osteoprotegerin, accumulate in patients with pore shrinkage syndrome, which may lead to an increased burden of WMHV [16,27]. Elevated levels of related inflammatory proteins in patients with atrophic foramen syndrome may also contribute to the development of hypertension, and these findings provide mechanistic insights from an epidemiological perspective. Further investigation of the possible pathophysiological mechanisms linking eGFRdiff to WMHV burden is warranted.

This study has several strengths. First, this is a large prospective cohort study which has approximately 36,000 participates. Second, we performed statistical adjustments for various covariates and a series of sensitivity analyses to ensure the robustness of the results. Third, we also explored for the first time the mediating role of systolic blood pressure, diastolic blood pressure and hypertension in the association between eGFRdiff and WMHV.

Our study has certain limitations. First, the prospective cohort of UK Biobank mainly consisted of white people who were more likely to have higher socioeconomic status and health consciousness. In addition, the small sample size in non-white groups led to unstable effect estimates, which made the association results in non-white groups less precise.Second, although adjusting for potential confounders, we can’t completely rule out residual confounding factors. Third, this study is limited by the narrow age range of participants (40–69 years), and further studies covering a broader age range are warranted.

## 5 Conclusion

Lower eGFR difference (eGFRdiff) was independently associated with increased white matter hyperintensity (WMHV). Our findings suggest that monitoring eGFRdiff could offer valuable insights for identifying the burden of cerebral small vessel disease in the general population, with potential implications for future clinical practice.

## Consent for publication

All authors read the manuscript and agreed to its publication.

## Ethics approval and consent to participate

The UK Biobank study was approved by the North West Multicentre Research Ethics Committee (REC reference: 21/NW/0157). All participants have provided written informed consent.

## Funding

This study was supported by 10.13039/501100001809National Natural Science Foundation of China (No. 82071337), Science and Technology Innovation Team of Anhui Province (No. 2024AH010014), and Research Fund of Anhui Institute of Translational Medicine (No. 2022zhyx-C38).

## Data availability statement

The UK Biobank data are available online at https://www.ukbiobank.ac.uk. All qualified researchers are able to apply for data used for the health-related research.

## CRediT authorship contribution statement

**QL and ZML:** study conceptualization; **ZML, FW and JCL:** manuscript drafting; **BBX:** visualization; **ZJW:** statistical analysis; **QL:** manuscript revision for intellectual contents and funding acquisition; All authors contributed to data interpretation and manuscript revision.

## Declaration of competing interest

The authors declare that they have no known competing financial interests or personal relationships that could have appeared to influence the work reported in this paper.

## Appendix Supplementary materials

Image, application 1
