# Supplementary material for: Association of the difference between cystatin C- and creatinine-based estimated glomerular filtration rate with cerebral small vessel disease: A large prospective cohort study
Source: J Prev Alzheimers Dis. 2025 Apr 24;12(7):100190. doi: 10.1016/j.tjpad.2025.100190 (PMC12321633; doi:10.1016/j.tjpad.2025.100190)
Supplement: Supplementary file 1 [file mmc1.docx]

| Supplementary Table 1. Association between eGFR difference and risk of Cerebral Small Vessel Disease, further adjusting for muscle mass. | | | | | | | | | | | | |
| --- | --- | --- | --- | --- | --- | --- | --- | --- | --- | --- | --- | --- |
|  |  |  |  |  |  |  | Group |  |  |  |  |  |
|  |  |  | Model1 |  |  |  | Model2 |  |  |  | Model3 |  |
| Characteristic |  | Beta | 95%CI^1^ | p-value |  | Beta | 95%CI^1^ | p-value |  | Beta | 95%CI^1^ | p-value |
| **eGFRabdiff** |  |  |  |  |  |  |  |  |  |  |  |  |
| -15~15 |  | 1.00 (Ref.) |  |  |  | 1.00 (Ref.) |  |  |  | 1.00 (Ref.) |  |  |
| <-15 |  | 0.14 | 0.11, 0.17 | <0.001 |  | 0.10 | 0.08, 0.13 | <0.001 |  | 0.08 | 0.05, 0.10 | <0.001 |
| >15 |  | -0.16 | -0.20, -0.12 | <0.001 |  | -0.06 | -0.10, -0.03 | <0.001 |  | -0.06 | -0.10, -0.03 | <0.001 |
| **eGFRrediff** |  |  |  |  |  |  |  |  |  |  |  |  |
| ≥0.7 |  | 1.00 (Ref.) |  |  |  | 1.00 (Ref.) |  |  |  | 1.00 (Ref.) |  |  |
| <0.7 |  | 0.33 | 0.26, 0.40 | <0.001 |  | 0.18 | 0.11, 0.24 | <0.001 |  | 0.09 | 0.02, 0.16 | 0.008 |

^1^CI = Confidence Interval

Note: data on ASM/BMI are available for 36849 participants.

Abbreviations: eGFRabdiff, absolute difference between cystatin- and creatinine-based estimated glomerular filtration rate; eGFRrediff, relative

difference between cystatin- and creatinine-based estimated glomerular filtration rate; ASM, appendicular skeletal muscle mass; BMI, body mass index.

Model 1: non-adjusted model.

Model 2: adjusted for age, gender, race and TDI.

Model 3: Model 2 plus obesity, smoking status, drinking status, diabetes, hypertension, hypercholesterolemia, TC, HDL-C, CRP, eGFRcys and ASM/BMI.

| Supplementary Table 2. Association between eGFR difference and risk of Cerebral Small Vessel Disease, further adjusting for eGFRcr and eGFRcr-cys. | | | | | | | | | | | | |
| --- | --- | --- | --- | --- | --- | --- | --- | --- | --- | --- | --- | --- |
|  |  |  |  |  |  |  | Group |  |  |  |  |  |
|  |  | Further adjusted for eGFRcys | | |  | Further adjusted for eGFRcr | | |  | Further adjusted for eGFRcr-cys | | |
| Characteristic |  | Beta | 95%CI^1^ | p-value |  | Beta | 95%CI^1^ | p-value |  | Beta | 95%CI^1^ | p-value |
| **eGFRabdiff** |  |  |  |  |  |  |  |  |  |  |  |  |
| -15~15 |  | 1.00 (Ref.) |  |  |  | 1.00 (Ref.) |  |  |  | 1.00 (Ref.) |  |  |
| <-15 |  | 0.07 | 0.04, 0.10 | <0.001 |  | 0.06 | 0.03, 0.08 | <0.001 |  | 0.04 | 0.02, 0.07 | <0.001 |
| >15 |  | -0.05 | -0.09, -0.02 | 0.003 |  | -0.03 | -0.06, 0.01 | 0.114 |  | -0.01 | -0.05, 0.03 | 0.602 |
| **eGFRrediff** |  |  |  |  |  |  |  |  |  |  |  |  |
| ≥0.7 |  | 1.00 (Ref.) |  |  |  | 1.00 (Ref.) |  |  |  | 1.00 (Ref.) |  |  |
| <0.7 |  | 0.08 | 0.01, 0.15 | 0.018 |  | 0.09 | 0.02, 0.15 | 0.008 |  | 0.06 | 0.00, 0.13 | 0.059 |

^1^CI = Confidence Interval

Abbreviations: eGFRabdiff, absolute difference between cystatin- and creatinine-based estimated glomerular filtration rate; eGFRrediff, relative

difference between cystatin- and creatinine-based estimated glomerular filtration rate.

All three groups are adjusted for age, gender, race and TDI, obesity, smoking status, drinking status, diabetes, hypertension, hypercholesterolemia, TC, HDL-C and CRP. Based on this, three groups were again adjusted separately for eGFRcys, eGFRcr and eGFRcr-cys.

| Supplementary Table 3. Association between eGFRrediff and risk of Cerebral Small Vessel Disease, when eGFRrediff is delimit at 0.6 | | | | | | | | | | | | |
| --- | --- | --- | --- | --- | --- | --- | --- | --- | --- | --- | --- | --- |
|  |  |  |  |  |  |  | Group |  |  |  |  |  |
|  |  |  | Model1 |  |  |  | Model2 |  |  |  | Model3 |  |
| Characteristic |  | Beta | 95%CI^1^ | p-value |  | Beta | 95%CI^1^ | p-value |  | Beta | 95%CI^1^ | p-value |
| **eGFRrediff** |  |  |  |  |  |  |  |  |  |  |  |  |
| ≥0.7 |  | 1.00 (Ref.) |  |  |  | 1.00 (Ref.) |  |  |  | 1.00 (Ref.) |  |  |
| <0.7 |  | 0.32 | 0.25, 0.40 | <0.001 |  | 0.18 | 0.11, 0.24 | <0.001 |  | 0.08 | 0.01, 0.15 | 0.018 |
| **eGFRrediff** |  |  |  |  |  |  |  |  |  |  |  |  |
| ≥0.6 |  | 1.00 (Ref.) |  |  |  | 1.00 (Ref.) |  |  |  | 1.00 (Ref.) |  |  |
| <0.6 |  | 0.56 | 0.35, 0.77 | <0.001 |  | 0.33 | 0.15, 0.51 | <0.001 |  | 0.22 | 0.04, 0.41 | 0.015 |

^1^CI = Confidence Interval

Abbreviations: eGFRrediff, relative difference between cystatin- and creatinine-based estimated glomerular filtration rate.

Model 1: non-adjusted model.

Model 2: adjusted for age, gender, race and TDI.

Model 3: Model 2 plus obesity, smoking status, drinking status, diabetes, hypertension, hypercholesterolemia, TC, HDL-C, CRP and eGFRcys.

| Supplementary Table 4. Association between eGFR difference and risk of Cerebral Small Vessel Disease after excluding patients with chronic kidney disease. | | | | | | | | | | | | |
| --- | --- | --- | --- | --- | --- | --- | --- | --- | --- | --- | --- | --- |
|  |  |  |  |  |  |  | Group |  |  |  |  |  |
|  |  |  | Model1 |  |  |  | Model2 |  |  |  | Model3 |  |
| Characteristic |  | Beta | 95%CI^1^ | p-value |  | Beta | 95%CI^1^ | p-value |  | Beta | 95%CI^1^ | p-value |
| **eGFRabdiff** |  |  |  |  |  |  |  |  |  |  |  |  |
| -15~15 |  | 1.00 (Ref.) |  |  |  | 1.00 (Ref.) |  |  |  | 1.00 (Ref.) |  |  |
| <-15 |  | 0.12 | 0.09, 0.15 | <0.001 |  | 0.10 | 0.07, 0.12 | <0.001 |  | 0.07 | 0.04, 0.10 | <0.001 |
| >15 |  | -0.17 | -0.21, -0.13 | <0.001 |  | -0.07 | -0.10, -0.03 | <0.001 |  | -0.06 | -0.10, -0.02 | 0.002 |
| **eGFRrediff** |  |  |  |  |  |  |  |  |  |  |  |  |
| ≥0.7 |  | 1.00 (Ref.) |  |  |  | 1.00 (Ref.) |  |  |  | 1.00 (Ref.) |  |  |
| <0.7 |  | 0.22 | 0.13, 0.30 | <0.001 |  | 0.14 | 0.07, 0.21 | <0.001 |  | 0.05 | -0.03, 0.12 | 0.227 |

^1^CI = Confidence Interval

Abbreviations: eGFRrediff, relative difference between cystatin- and creatinine-based estimated glomerular filtration rate.

Model 1: non-adjusted model.

Model 2: adjusted for age, gender, race and TDI.

Model 3: Model 2 plus obesity, smoking status, drinking status, diabetes, hypertension, hypercholesterolemia, TC, HDL-C, CRP and eGFRcys.

Chronic kidney disease was defined as an eGFRcys or eGFRcr of less than 60 ml/ minute /1.73m2

| Supplementary Table 5. Association between eGFR difference based on eGFR measured in 2012-2014 and risk of Cerebral Small Vessel Disease | | | | | | | | | | | | |
| --- | --- | --- | --- | --- | --- | --- | --- | --- | --- | --- | --- | --- |
|  |  |  |  |  |  |  | Group |  |  |  |  |  |
|  |  |  | Model1 |  |  |  | Model2 |  |  |  | Model3 |  |
| Characteristic |  | Beta | 95%CI^1^ | p-value |  | Beta | 95%CI^1^ | p-value |  | Beta | 95%CI^1^ | p-value |
| **eGFRabdiff** |  |  |  |  |  |  |  |  |  |  |  |  |
| -15~15 |  | 1.00 (Ref.) |  |  |  | 1.00 (Ref.) |  |  |  | 1.00 (Ref.) |  |  |
| <-15 |  | 0.14 | 0.06, 0.22 | <0.001 |  | 0.11 | 0.04, 0.18 | 0.001 |  | 0.07 | 0.00, 0.14 | 0.044 |
| >15 |  | -0.05 | -0.15, 0.06 | 0.366 |  | 0.01 | -0.08, 0.10 | 0.864 |  | 0.02 | -0.07, 0.11 | 0.658 |
| **eGFRrediff** |  |  |  |  |  |  |  |  |  |  |  |  |
| ≥0.7 |  | 1.00 (Ref.) |  |  |  | 1.00 (Ref.) |  |  |  | 1.00 (Ref.) |  |  |
| <0.7 |  | 0.35 | 0.15, 0.54 | <0.001 |  | 0.29 | 0.12, 0.46 | <0.001 |  | 0.20 | 0.03, 0.37 | 0.023 |

^1^CI = Confidence Interval

Abbreviations: eGFRrediff, relative difference between cystatin- and creatinine-based estimated glomerular filtration rate.

Model 1: non-adjusted model.

Model 2: adjusted for age, gender, race and TDI.

Model 3: Model 2 plus obesity, smoking status, drinking status, diabetes, hypertension, hypercholesterolemia, TC, HDL-C, CRP and eGFRcys.
